# Supplementary material for: Receptor-specific interactome as a hub for rapid cue-induced selective translation in axons
Source: eLife. 2019 Nov 20;8:e48718. doi: 10.7554/eLife.48718 (PMC6894925; doi:10.7554/eLife.48718)
Supplement: Transparent reporting form [file elife-48718-transrepform.docx]

***eLife’s* transparent reporting form**

We encourage authors to provide detailed information *within their submission* to facilitate the interpretation and replication of experiments. Authors can upload supporting documentation to indicate the use of appropriate reporting guidelines for health-related research (see [EQUATOR Network](http://www.equator-network.org/%20)), life science research (see the [BioSharing Information Resource](https://biosharing.org/)), or the [ARRIVE guidelines](http://www.plosbiology.org/article/info:doi/10.1371/journal.pbio.1000412) for reporting work involving animal research. Where applicable, authors should refer to any relevant reporting standards documents in this form.

If you have any questions, please consult our Journal Policies and/or contact us: [editorial@elifesciences.org](mailto:editorial@elifesciences.org).

**Sample-size estimation**

- You should state whether an appropriate sample size was computed when the study was being designed
- You should state the statistical method of sample size computation and any required assumptions
- If no explicit power analysis was used, you should describe how you decided what sample (replicate) size (number) to use

Please outline where this information can be found within the submission (e.g., sections or figure legends), or explain why this information doesn’t apply to your submission:

We did not perform an explicit power analysis. The sample size and number of replicates for IP-mass-spectrometry, IP-RNA-sequencing data, qPCR and Western blotting was based on previous literature. The sample size and number of replicates for all imaging data (PLA, quantitative immunofluorescence) was based on previous studies published by the Holt lab and others using RGC axonal growth cones.

**Replicates**

- You should report how often each experiment was performed
- You should include a definition of biological versus technical replication
- The data obtained should be provided and sufficient information should be provided to indicate the number of independent biological and/or technical replicates
- If you encountered any outliers, you should describe how these were handled
- Criteria for exclusion/inclusion of data should be clearly stated
- High-throughput sequence data should be uploaded before submission, with a private link for reviewers provided (these are available from both GEO and ArrayExpress)

Please outline where this information can be found within the submission (e.g., sections or figure legends), or explain why this information doesn’t apply to your submission:

The number of experiments (biological replicates) are indicated in the figure legends and/or materials and methods section. Each experiment that was quantified was repeated at least three times. For qPCR experiments, at least three biological replicates with each of them three technical replicates were performed. Criteria for inclusion of data (in case of QIF, Expansion microscopy and PLA) is indicated in the methods section where it is described how growth cones are (randomly) selected for quantification.

RNA-sequencing data associated with this manuscript has been deposited on the GEO database (identifier GSE135338). All proteomics data associated with this manuscript has been uploaded to the PRIDE online repository (identifier: PXD015650).

**Statistical reporting**

- Statistical analysis methods should be described and justified
- Raw data should be presented in figures whenever informative to do so (typically when N per group is less than 10)
- For each experiment, you should identify the statistical tests used, exact values of N, definitions of center, methods of multiple test correction, and dispersion and precision measures (e.g., mean, median, SD, SEM, confidence intervals; and, for the major substantive results, a measure of effect size (e.g., Pearson's r, Cohen's d)
- Report exact p-values wherever possible alongside the summary statistics and 95% confidence intervals. These should be reported for all key questions and not only when the p-value is less than 0.05.

Please outline where this information can be found within the submission (e.g., sections or figure legends), or explain why this information doesn’t apply to your submission:

All experiments were performed in at least three independent biological replicates unless explicitly stated otherwise. The order of data collection was randomized, and no data were excluded from analysis. Statistical analysis was performed using GraphPad Prism, R or MATLAB. All the information on statistical tests used, exact values of n and dispersion and precision measures and p values are described in the figure legends and/or the methods section. The number of datapoints are indicated in the figure graphs for each quantitative experiment.

(For large datasets, or papers with a very large number of statistical tests, you may upload a single table file with tests, Ns, etc., with reference to sections in the manuscript.)

**Group allocation**

- Indicate how samples were allocated into experimental groups (in the case of clinical studies, please specify allocation to treatment method); if randomization was used, please also state if restricted randomization was applied
- Indicate if masking was used during group allocation, data collection and/or data analysis

Please outline where this information can be found within the submission (e.g., sections or figure legends), or explain why this information doesn’t apply to your submission:

Group allocation does not apply to this study.

**Additional data files (“source data”)**

- We encourage you to upload relevant additional data files, such as numerical data that are represented as a graph in a figure, or as a summary table
- Where provided, these should be in the most useful format, and they can be uploaded as “Source data” files linked to a main figure or table
- Include model definition files including the full list of parameters used
- Include code used for data analysis (e.g., R, MatLab)
- Avoid stating that data files are “available upon request”

Please indicate the figures or tables for which source data files have been provided:

Not applicable.

The source data for RNA-sequencing and proteomics have been uploaded to GEO (GSE135338) and PRIDE(PXD015650) databases. Analyses obtained from these data are displayed in Figure 1, Figure 2 and Figure 2 – Figure Supplement 1.

Source data for all immunocytochemistry and PLA experiments have been provided and correspond to Figure 2, Figure 3, Figure 3 – Figure Supplement 3, Figure 4 and Figure 4 – Figure Supplement 4. In these excel files it is indicated for each tab to which figure panel the data corresponds.
